# Supplementary material for: Dissecting reversible and irreversible single cell state transitions from gene regulatory networks
Source: Mol Syst Biol. 2026 Feb 9;22(5):811–32. doi: 10.1038/s44320-026-00196-8 (PMC13144439; doi:10.1038/s44320-026-00196-8)
Supplement: Supplementary file 2 — Appendix [file 44320_2026_196_MOESM2_ESM.pdf]

Appendix for “Dissecting reversible and irreversible single cell state transitions from gene regulatory networks”

Daniel A. Ramirez and Mingyang Lu

Table of Contents

Appendix Figures *3*

Appendix Figure S1: Density plots, clustering and gene expression heatmaps for synthetic circuits........ 3

Appendix Figure S2. Time trajectory for incoherent feedforward loop (iFFL) simulation. 4

Appendix Figure S3: Comparison between STICCC and RNA velocity methods. 5

Appendix Figure S4. Vector fields for coupled toggle switch (CTS) and repressilator (REP) circuits at low and high levels of simulated noise. 6

Appendix Figure S5: CTS basin positions during simulated perturbation........ 7

Appendix Figure S6. Edge sensitivity analysis reveals roles and importance of network edges. 8

Appendix Figure S7: Edge ablation analysis for simulated synthetic circuits........ 9

Appendix Figure S8. Selection of lag periods for time trajectory comparisons. 10

Appendix Figure S9: Computational time cost benchmarking. 11

Appendix Tables........*12*

Appendix Table S1. Parameters for the incoherent feedforward loop (iFFL) trajectory simulation........12

Appendix Table S2. Parameters for stochastic simulation of the repressilator (REP) circuit. 13

Appendix Table S3. Parameters for stochastic simulation of the coupled toggle switch (CTS) circuit. 14

Appendix Table S4. Network node mappings for the budding yeast cell cycle GRN. 15

Appendix Table S5. Network node mappings for the mammalian cell cycle GRN. 16

Appendix Table S6. GRN used for hematopoietic stem cell dataset........17

Appendix Table S7. Parameters and input gene expression space used for STICCC on synthetic and experimental circuits. 18

Appendix Figures

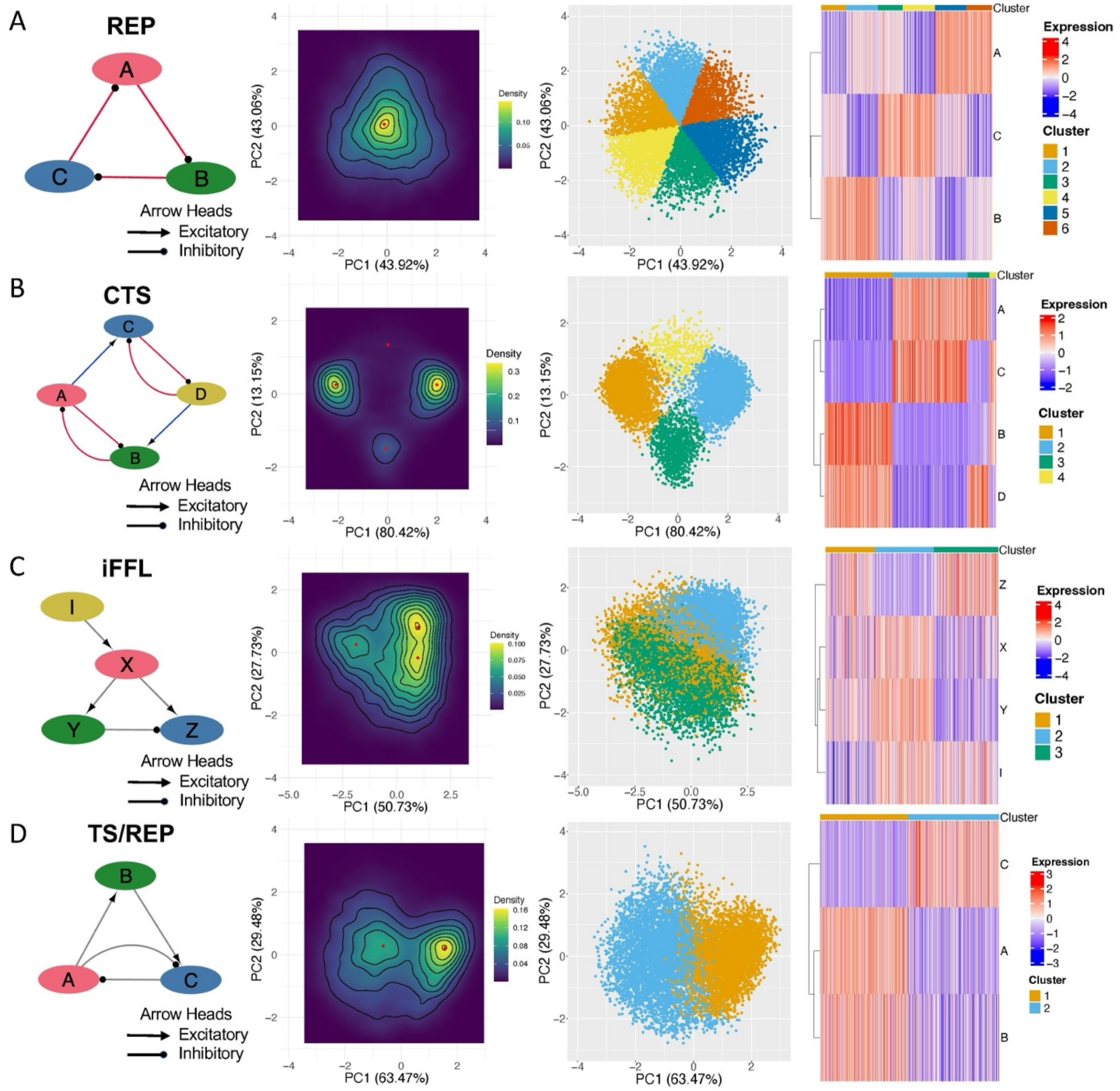

Appendix Figure S1: Density plots, clustering and gene expression heatmaps for synthetic circuits. Leftmost column shows circuit topologies. Second column shows 2D density heatmaps with contour lines, with red points indicating the basin positions as identified by local peaks in density. Third column shows PCA projections of RACIPE-simulated gene expression data with points colored by cluster. Fourth column shows gene expression heatmaps for simulated data, with genes in rows and models in columns. Columns are grouped by cluster. Results are shown for **A)** repressilator (REP), **B)** coupled toggle switch (CTS), **C)** incoherent Feedforward loop (iFFL), and **D)** toggle switch/repressilator (TS/REP) circuits.

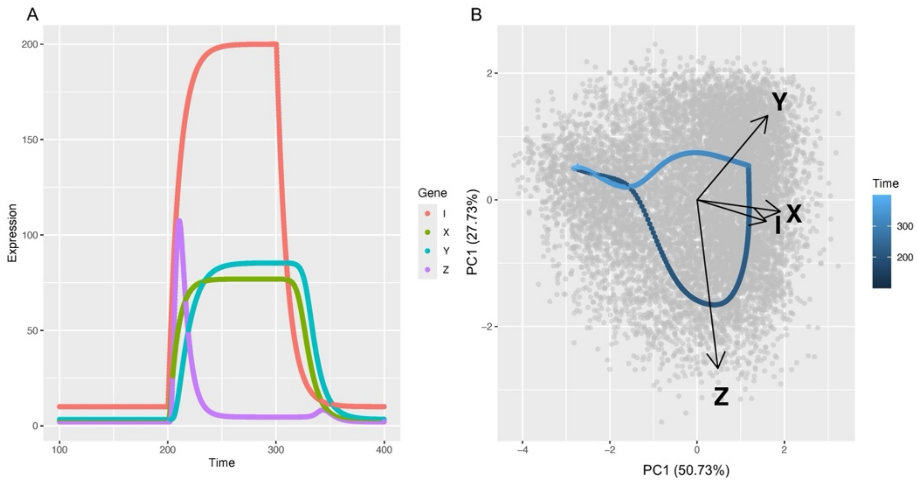

Appendix Figure S2. Time trajectory for incoherent feedforward loop (iFFL) simulation. A model with parameters as described in **Table S1** was simulated deterministically with an increase in production rate for signal node I at t=200, which was then removed at t=300. **A)** Line plot of expression values over time for each gene in the network, colored by gene. **B)** Time trajectory from (A) projected to the first two principal components from ensemble simulation of the FFL circuit (by RACIPE), with ensemble data shown in grey and the trajectory plotted on top, colored by time (dark blue to light blue). Loading vectors for each gene in the first two principal components are also plotted and labeled with arrows.

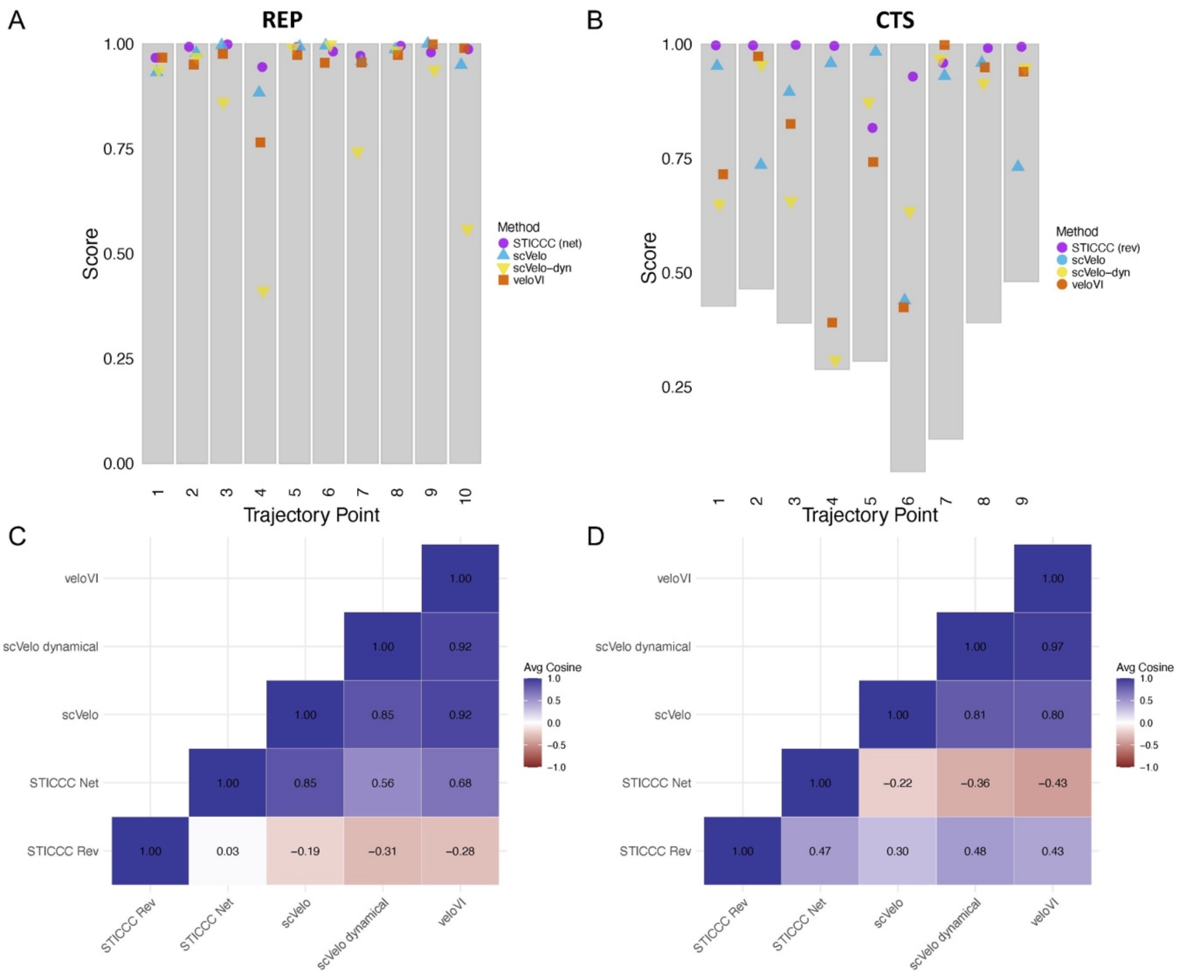

Appendix Figure S3: Comparison between STICCC and RNA velocity methods. A-B) Point-wise cosine similarity between predicted vectors from various methods and the most likely observed vectors for simulated cells along the REP deterministic limit cycle **(A)** and path between states in CTS **(B)**. Method is denoted by point color and shape. Background grey bars indicate the theoretical range of scores for each point. **C)** Heatmap of average cosine similarity of paired vectors from a simulated repressilator (REP) dataset between STICCC net flow (Net), reversibility (Rev), and three implementations of RNA velocity algorithms. Cosine similarity is indicated by color. **D)** Heatmap of average cosine similarity of paired vectors between STICCC and RNA velocity methods for coupled toggle switch (CTS) dataset.

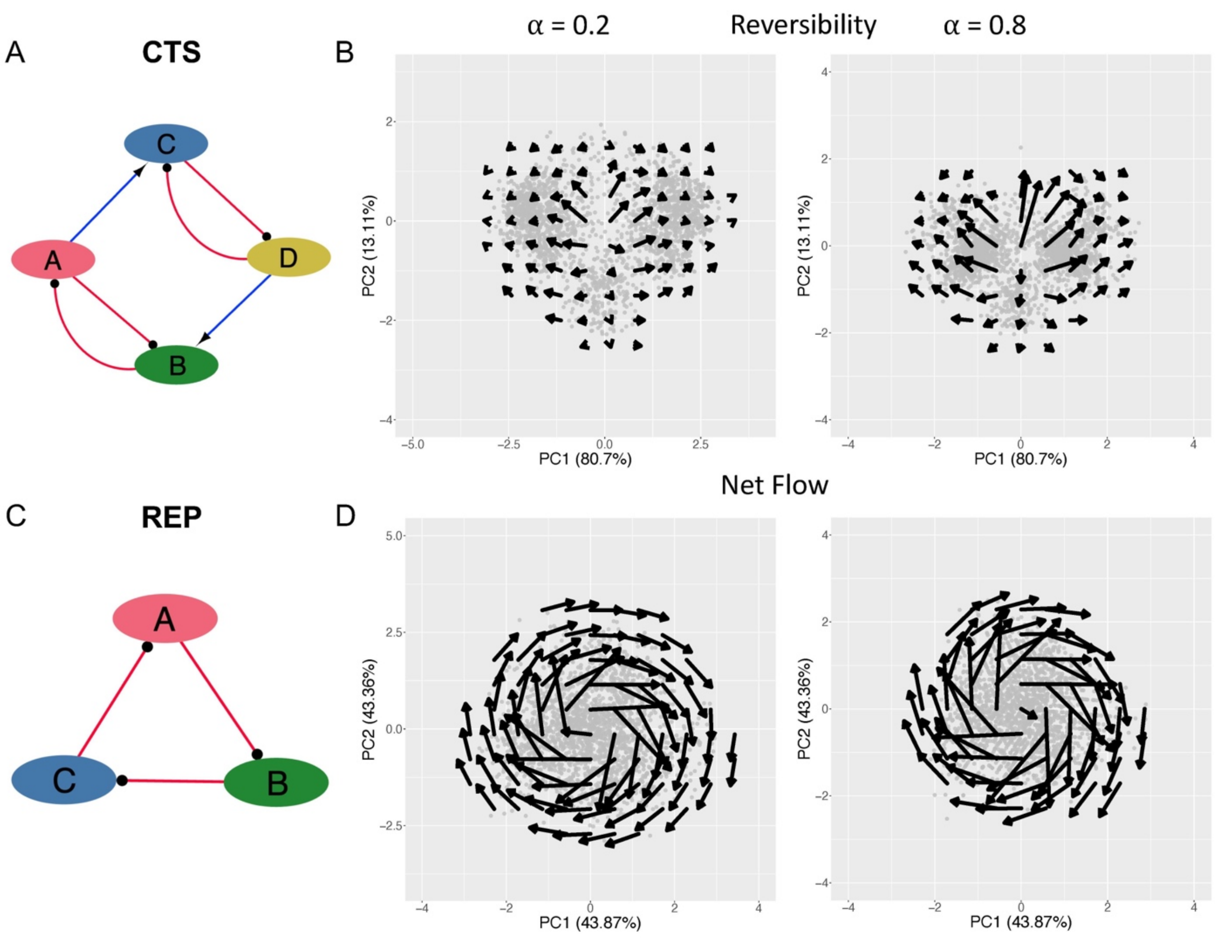

Appendix Figure S4. Vector fields for coupled toggle switch (CTS) and repressilator (REP) circuits at low and high levels of simulated noise. A) CTS topology diagram. **B)** Reversibility vector field for CTS at low (a = 0.2) and high (a = 0.8) dropout levels. **C)** REP topology diagram. **D)** Net flow vector field for REP at low and high dropout levels.

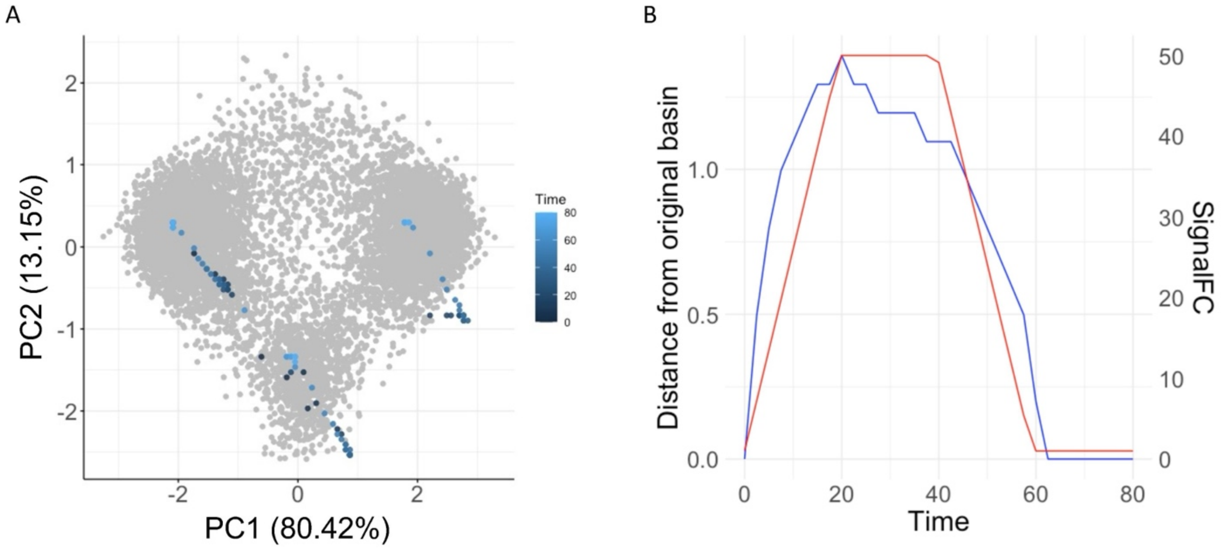

Appendix Figure S5: CTS basin positions during simulated perturbation. A) PCA projection of CTS simulated gene expression (from ensemble simulation by RACIPE) in grey, with basins identified by density overlaid in blue. Basin positions are colored according to the time during induced transition simulations. **B)** Line plot of signal strength (red), in terms of fold change, and Euclidean distance from original basin position in PCA space (blue) for the leftmost basin in simulated data over time.

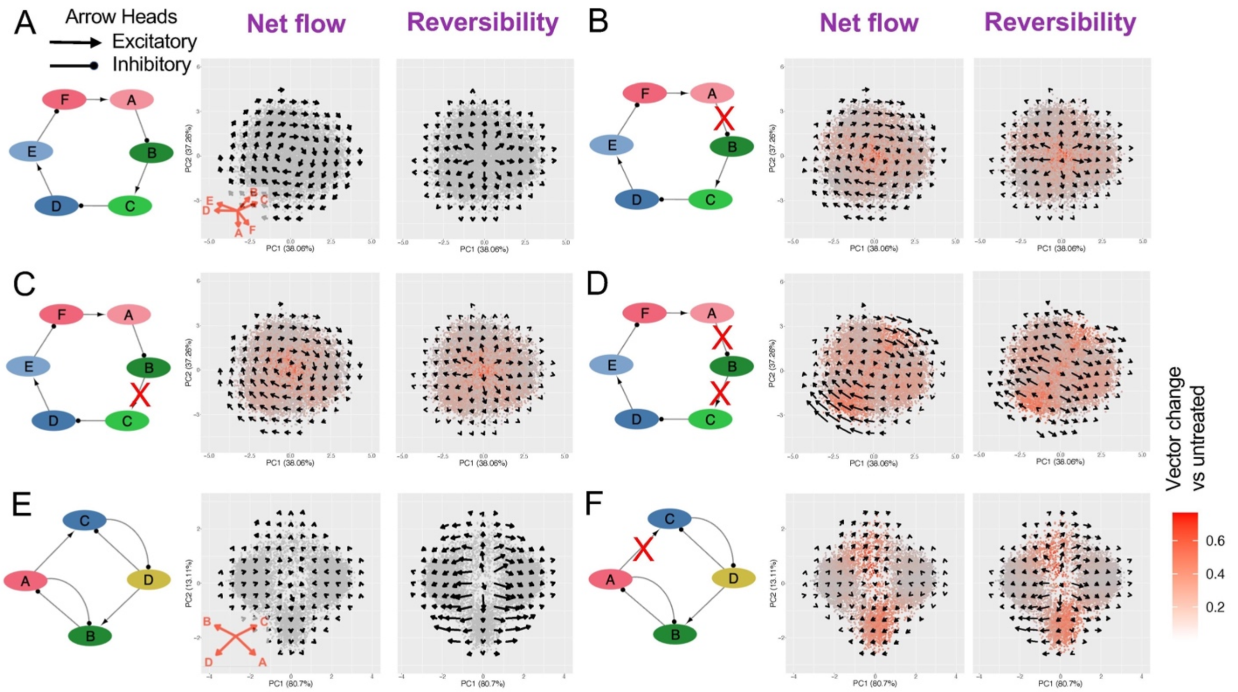

Appendix Figure S6. Edge sensitivity analysis reveals roles and importance of network edges. Using simulations of a 6-gene repressilator (REP) and (coupled toggle switch) CTS, different subsets of the true topology were provided to STICCC to infer edge importance based on the resulting change in vectors. Point color denotes relative change in vector prediction. **A)** Vector predictions for the 6-gene REP with the full circuit topology. **B-D)** Predictions for the 6-gene REP with one or two edges omitted from the circuit topology. **E)** Predictions for CTS with the full circuit topology. **F)** Predictions for CTS with the edge from A to C omitted from the circuit topology.

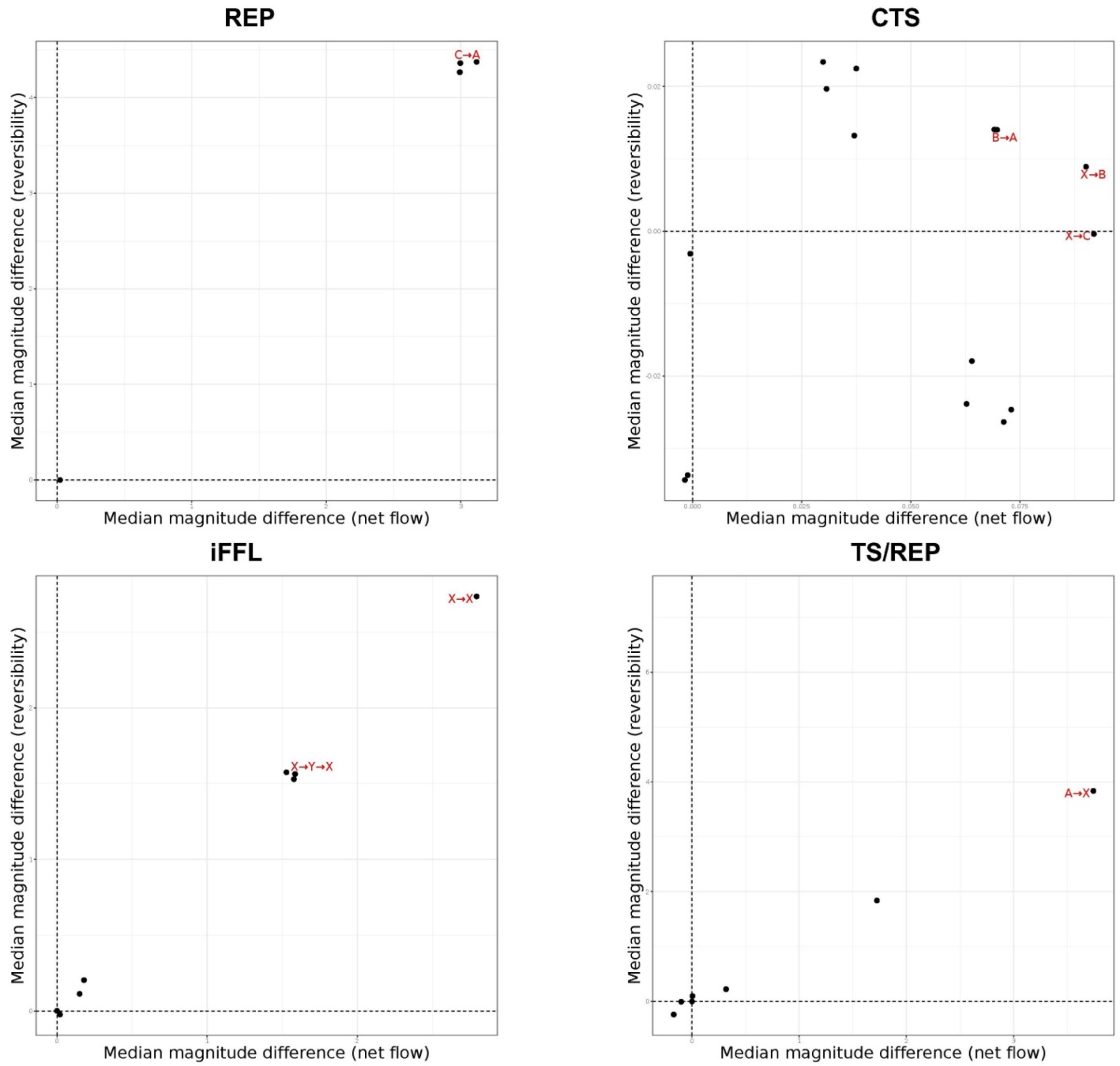

Appendix Figure S7: Edge ablation analysis for simulated synthetic circuits. Summary of edge sensitivity analysis showing median magnitude difference between paired vectors across edge perturbations, where vectors are computed with part of the GRN intentionally left out. X-axis shows the differences in net flow, and y-axis shows the differences in reversibility. Red labels highlight the perturbations with the largest changes (top 15% by combined mean difference) in either net flow or reversibility. Panels show results for simulated repressilator (REP), coupled toggle switch (CTS), incoherent feedforward loop (iFFL), and coupled toggle switch/repressilator (TS/REP) circuits respectively.

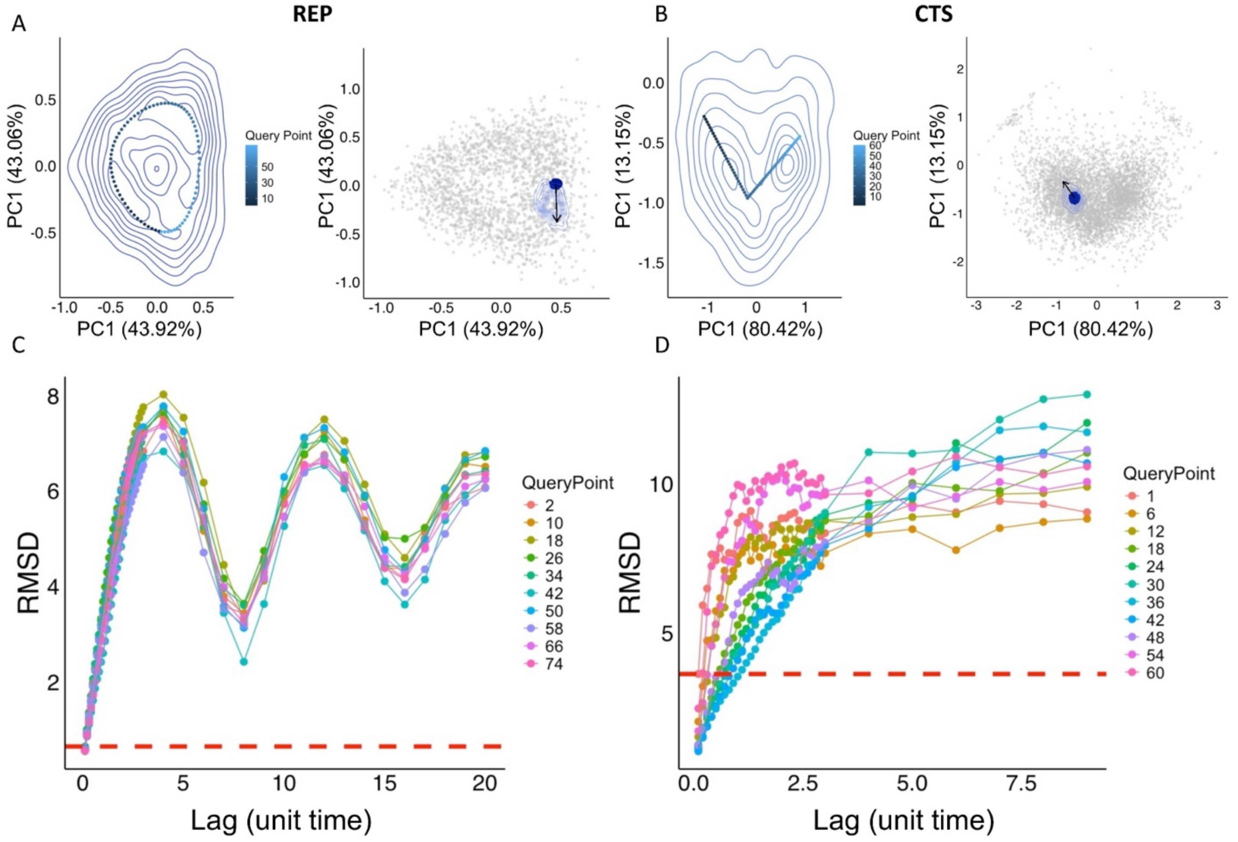

Appendix Figure S8. Selection of lag periods for time trajectory comparisons. A) Left panel: Density plot of gene expression snapshots from a noisy time trajectory simulation of the REP circuit with blue contour lines, projected onto the first two principal components from the simulated gene expression of an ensemble of 10000 models. Points shown are snapshots of the deterministic limit cycle, colored and indexed 1-77 with earlier timepoints in black and later timepoints in blue. Right panel: Illustrative example of points selected to compare with predicted angles. Points in dark blue are sampled from the noisy trajectory by proximity to a selected point from the deterministic cycle. Blue contour lines show the time evolution of each point in the previous group after a time lag of 0.5. Arrow shows the predicted v_1 vector for the selected point from the limit cycle. **B)** Left panel: Density plot of gene expression snapshots from a noisy time trajectory simulation of CTS circuit with blue contour lines, projected onto the first two principal components from the simulated gene expression of an ensemble of 10000 models. Points shown are a linear interpolation between the medians of three clusters identified with k-means clustering, colored and indexed 1-60 with earlier timepoints in black and later timepoints in blue. Right panel: Illustrative example of points selected to compare with predicted angles. Points in dark blue are sampled from the noisy trajectory by proximity to a selected point from the linear path. Blue contour lines show the time evolution of each point in the previous group after a time lag of 0.5. Arrow shows the predicted v_2 vector for the selected point from the linear path. **C)** Line plot of root mean square deviation (RMSD) between initial timepoints and final timepoints by lag period, for 10 points from the REP deterministic limit cycle (indicated by color). Target RMSD, selected based on the highest RMSD of any query point at a lag of 0.1, is indicated by a red horizontal dashed line. **D)** RMSD by lag time between initial and final timepoints for 11 points from the CTS linear transition path, indicated by color. Target RMSD is selected and drawn as in (C). Lag times for REP and CTS were selected independently for each point based on target RMSD; as such, the lag time for the analysis of REP in **Fig. 3C** was generally constant, whereas for CTS the lag time varies based on the position of the initial states.

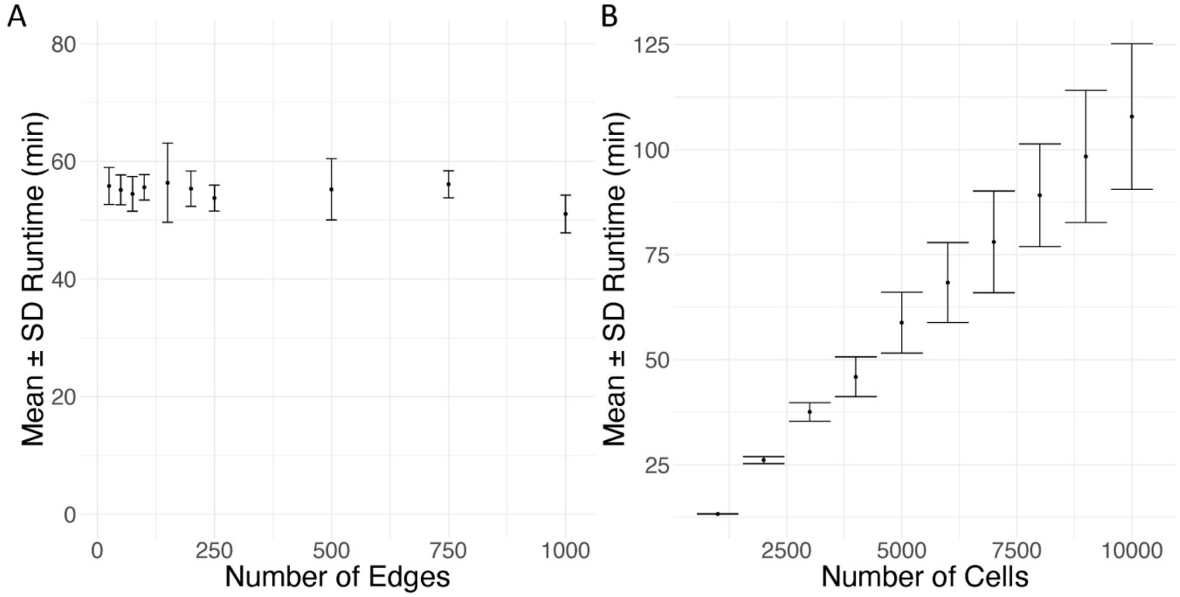

Appendix Figure S9: Computational time cost benchmarking. A) Mean and standard deviation of runtime across 5 trials for STICCC applied to a dataset of 3133 cells with GRNs inferred using GENIE3 at various sizes. **B)** Mean and standard deviation of runtime across 5 trials for STICCC applied to CTS circuit simulations with varying numbers of cells between 1000 and 10000. All benchmarks were performed on a compute node in the Northeastern University Explorer Cluster. Each job ran on a dual-socket Intel Xeon E5-2680 v2 CPU running a CentOS operating system.

Appendix Tables

Appendix Table S1. Parameters for the incoherent feedforward loop (iFFL) trajectory simulation. Simulations were conducted with the signal node I in the ‘off’ state for 200 time units, followed by 100 time units with the signal in the ‘on’ state, and a final 100 in the original state.

Parameter	Gene/Edge	Value
**Signal Production Rate, G**	I (off)	1
	I (on)	20
	X	10
	Y	10
	Z	30
**Degradation Rate, k**	I	0.1
	X	0.13
	Y	0.1
	Z	0.15
**Hill Coefficient, n**	All	4
**Fold Change, $\boldsymbol{\lambda}$**	I activates X	50
	X activates Y	30
	Y inhibits Z	50
	X activates Z	100
**Threshold, $\boldsymbol{X_0}$**	I activates X	30
	X activates Y	50
	Y inhibits Z	20
	X activates Z	20
**Simulation Time**	-	400

Appendix Table S2. Parameters for stochastic simulation of the repressilator (REP) circuit.

Parameter	Value (simulations without splicing)	Value (simulations with splicing)
Production Rate, G	30	40
Degradation Rate, k	0.5	0.2
Hill Coefficient, n	4	4
Fold Change, λ	50	50
Threshold, X_0	20	20
Simulation Time	10000	5000
Simulation Noise, ξ	0.1	0.05
Splicing Rate, β	–	0.7

Appendix Table S3. Parameters for stochastic simulation of the coupled toggle switch (CTS) circuit. Most parameters are constant for all genes and edges, except a modified threshold value for the edges from B to A and C to D, which increases the activity of these two edges.

Parameter	Value (simulations without splicing)	Value (simulations with splicing)
Production Rate, G	50	50
Degradation Rate, k	0.1	0.15
Hill Coefficient, n	4	4
Fold Change, λ	10	10
Threshold, X_0	100	100
Threshold (modified), X_{0M}	80	80
Simulation Time	100000	100000
Simulation Noise, ξ	2	0.04
Splicing Rate, β	–	0.7

Appendix Table S4. Network node mappings for the budding yeast cell cycle GRN. Shown are the systematic gene names, the standard names, and the corresponding network node names.

Systematic Name	Standard Name	Network Node Name
YAL040C	CLN3	Cln3
YAR007C	RFA1	MBF
YIL066C	RNR3	Whi5
YER111C	SWI4	SBF
YMR199W	CLN1	Cln1,2
YGR109C	CLB6	Clb5,6
YLR079W	SIC1	Sic1
YGL003C	CDH1	Cdh1
YDR225W	HTA1	DNA Synthesis (DNA-S)
YGR108W	CLB1	Clb1,2
YMR043W	MCM1	Mcm1
YGL116W	CDC20	Cdc20
YDR146C	SWI5	Swi5
YDR113C	PDS1	Pds1
YAR019C	CDC15	Cdc14

Appendix Table S5. Network node mappings for the mammalian cell cycle GRN.

Network Node	Gene used in STICCC
CycD/Cdk4	CCND1
p27	CDKN1B
E2F1	E2F1
Rb	RB1
CycE/Cdk2	CCNE1
CycA/Cdk2	CCNA1
DNA Replication	H2AX
Cdh1/APC	FZR1
Emi1	FBXO5
E2F7	E2F7
CycB/Cdk1	CCNB1
Cdc20/APC	CDC20
MCM	MCM2

Appendix Table S6. GRN used for hematopoietic stem cell dataset. Original network topology was obtained from prior work by Mojtahedi et al, 2016, in PLoS Biology 14(12), referenced in the main text. We removed genes with low measured variance (Runx1, cJun, C/EBPa, EpoR, Eklf, Egr-2, Gfi-1, Fli-1).

Source	Target	Type
GATA2	GATA2	Activation
GATA2	GATA1	Activation
GATA1	PU.1	Inhibition
PU.1	GATA1	Inhibition
GATA1	GATA1	Activation
PU.1	PU.1	Activation
GATA1	Fog-1	Activation
PU.1	Scl	Inhibition
GATA1	Scl	Activation
GATA1	c-Kit	Inhibition
Scl	c-Kit	Activation
Fog-1	GATA2	Inhibition
Fog-1	c-Myb	Inhibition
GATA2	PU.1	Inhibition
PU.1	GATA2	Inhibition
GATA1	c-Myb	Activation
GATA1	GATA2	Inhibition
GATA1	Hbaa1	Activation
c-Myb	GATA1	Inhibition
PU.1	CD11b	Activation
PU.1	Fog-1	Inhibition

Appendix Table S7. Parameters and input gene expression space used for STICCC on synthetic and experimental circuits.

Dataset	Type	Sampling Radius	Expression Space	Number of Models/Cells
REP	Simulated	0.05	All PCs	10000
CTS				
iFFL				
TS/REP				
REP - time series		0.15		2000
REP - down-sampled		0.1		200
REP - dropout		0.1		9994
CTS - time series		0.15		5000
CTS - dropout		0.2		2000
CTS - signaling		0.05		4377
Cell cycle (yeast)	Experimental	0.15	Top 10 PCs	976
Cell cycle (mammalian)		0.2	Top 10 PCs	1152
HSC		0.2	Top 5 PCs	1600
EMT		0.3	Top 15 PCs	3133
