# Supplementary material for: Dissecting reversible and irreversible single cell state transitions from gene regulatory networks
Source: Mol Syst Biol. 2026 Feb 9;22(5):811–32. doi: 10.1038/s44320-026-00196-8 (PMC13144439; doi:10.1038/s44320-026-00196-8)
Supplement: Supplementary file 3 — Expanded View Figures [file 44320_2026_196_MOESM3_ESM.pdf]

Expanded View Figures

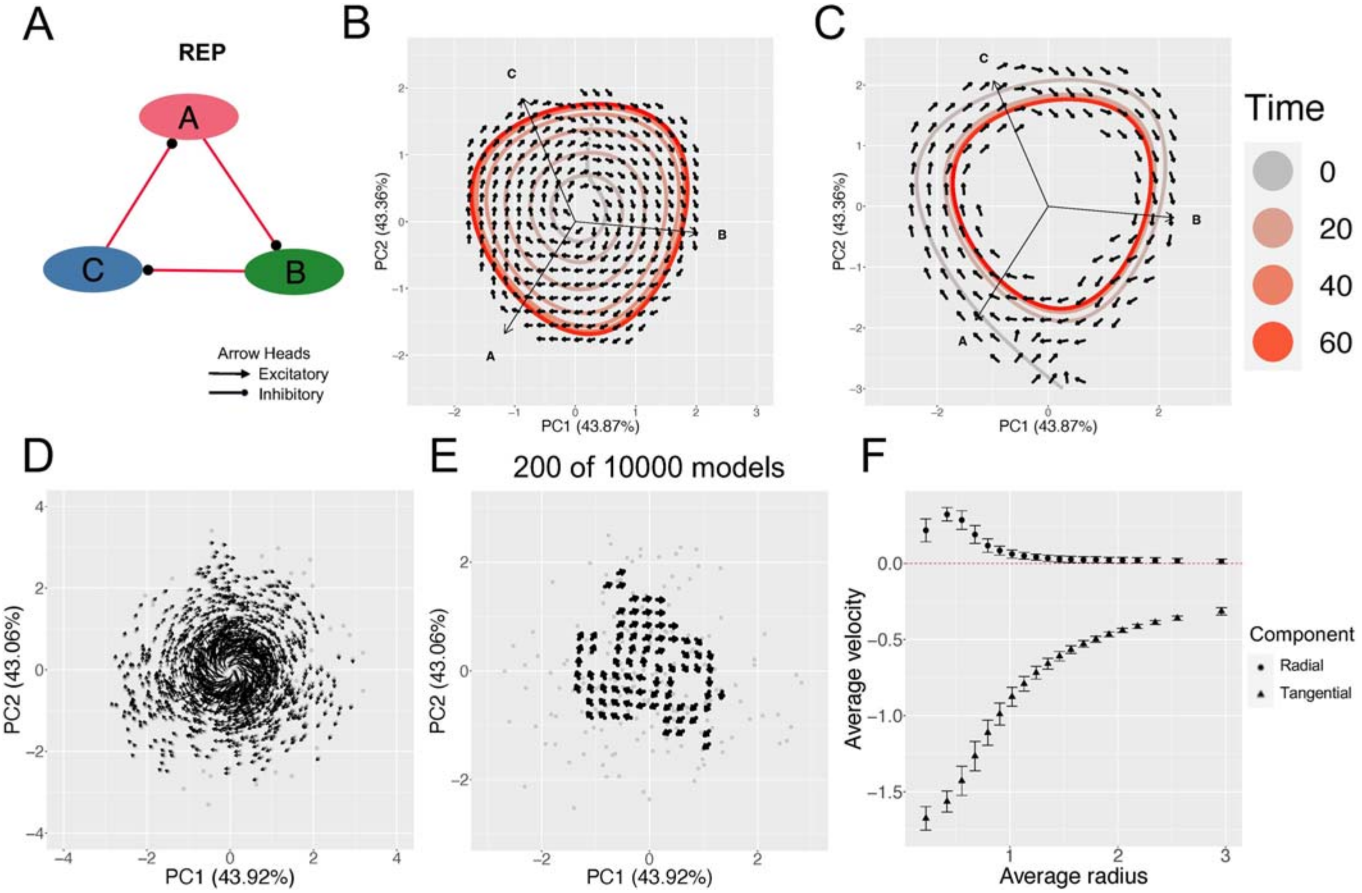

Figure EV1. Repressilator (REP) time trajectory simulations and additional analysis.

(**A**) REP circuit topology diagram. (**B**, **C**) STICCC predictions (vectors shown as arrows) using the gene expression snapshots from the simulated REP trajectories approaching the limit cycle (with later time points indicated in red) from two different initial conditions (with earlier time points indicated in gray). The plots show the projection of the time-series gene expression data onto the first two principal components from the simulated gene expression of an ensemble of 10,000 models. (**D**) Cell-specific outgoing transition vectors v_1 calculated for a simulated gene expression of an ensemble of 10,000 REP models. Shown is a random subset of 1000 cells and their predicted vectors. (**E**) State transition pattern is conserved even with extreme under-sampling. Grid-smoothed vector field for REP circuit is shown after calculating vectors on a subset of 200 out of 10,000 models. (**F**) Mean values of the radial (circles) and tangential (triangles) components of the inferred vectors for cells in various radial bands around the origin of the gene expression space, projected onto the first two principal components ($n = 500$ per x value). The inferred transition vectors are predominantly dominated by the tangential components, suggesting oscillatory state transitions. Error bars are drawn at $+/-$ 1 standard deviation.

 © The Author(s)

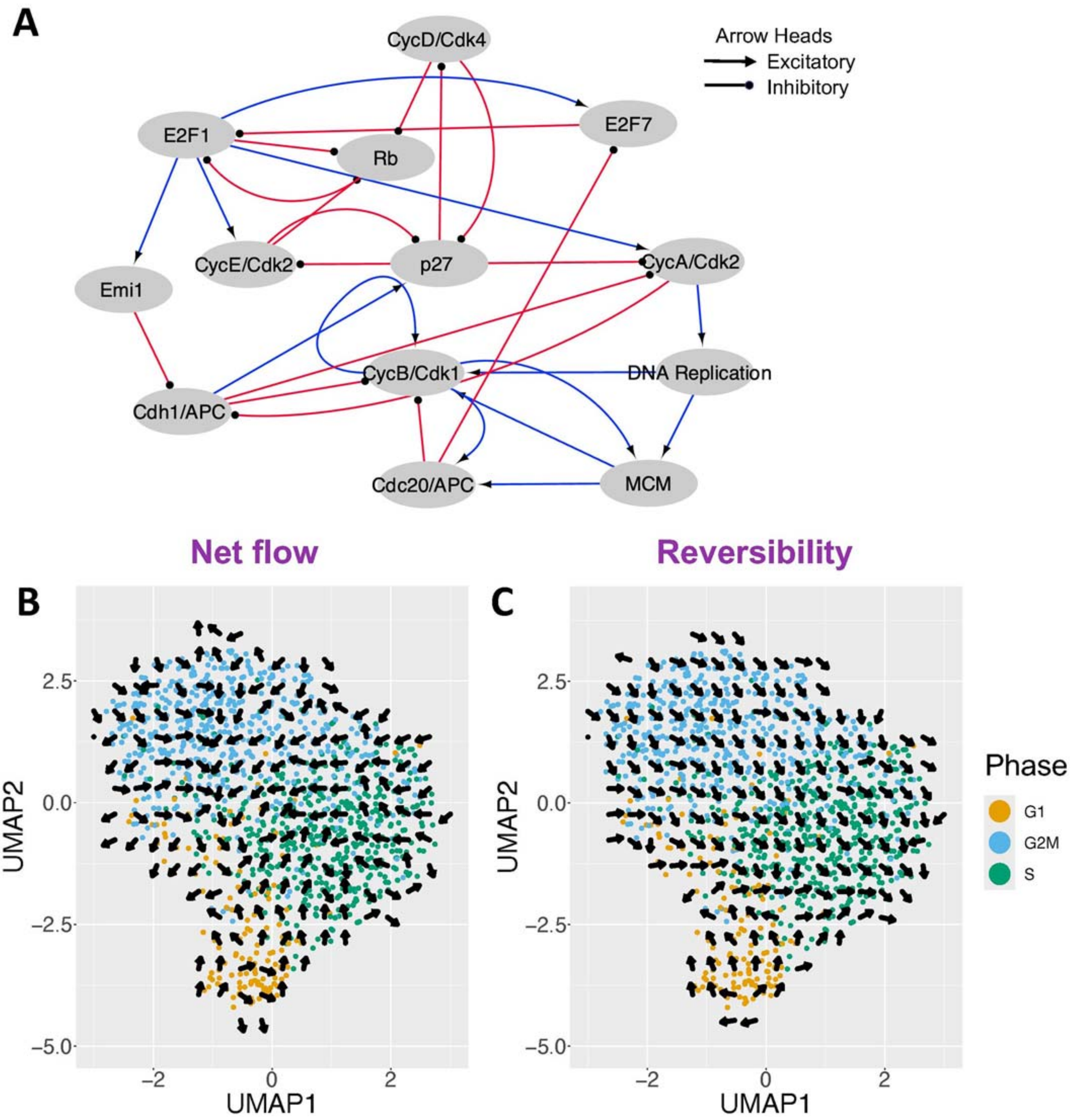

Figure EV2. STICCC predictions for U2OS cell cycle scRNA-seq data.

(**A**) Modified cell cycle circuit topology for mammalian systems. (**B**) UMAP projection of U2OS cells with STICCC net flow predictions. Points are colored by cell cycle phase. (**C**) UMAP projection of U2OS cells with STICCC reversibility predictions.

© The Author(s)

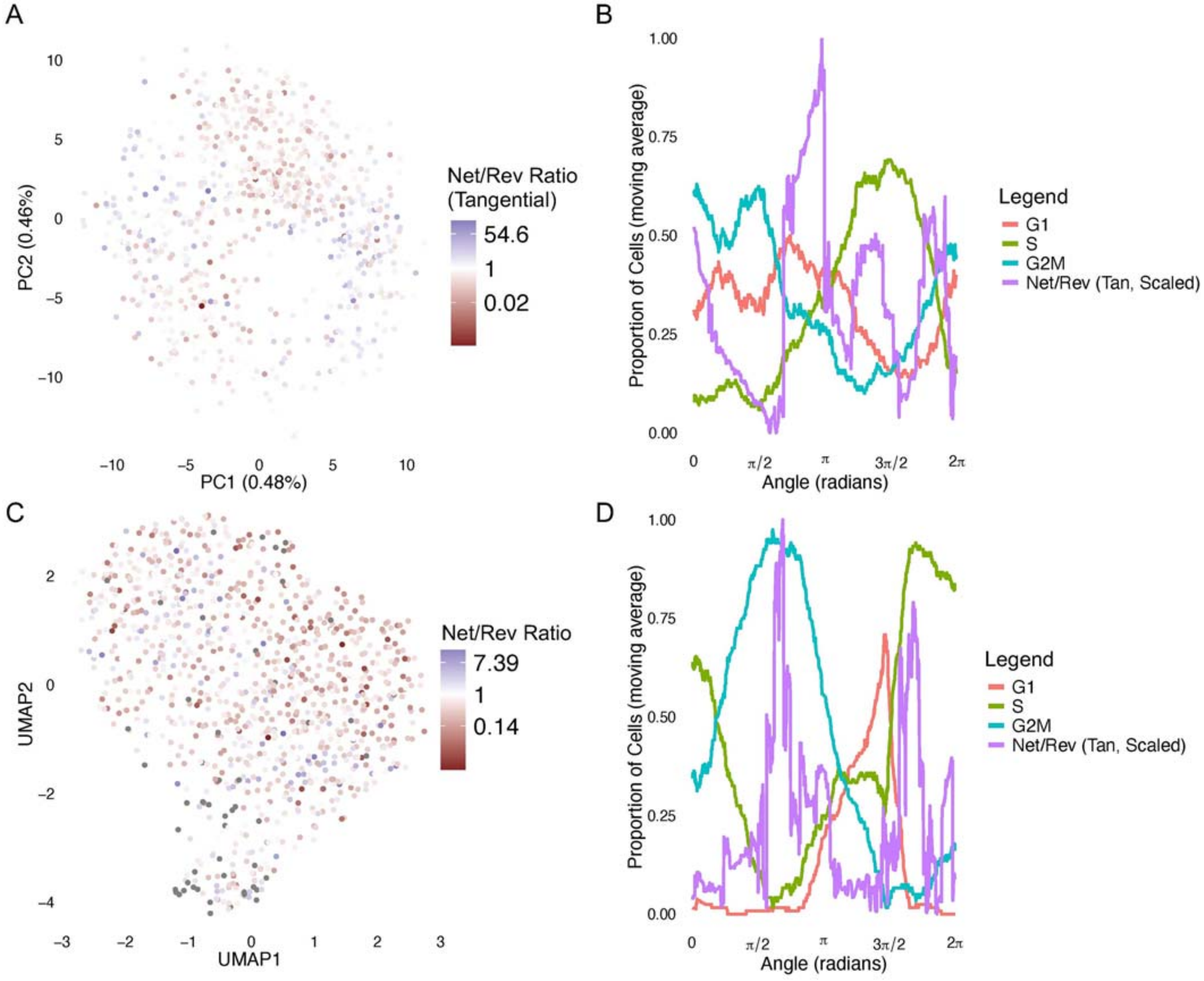

Figure EV3. Analysis of STICCC vectors along cell cycle phases.

(**A**) PCA projection of yeast scRNA-seq data colored by the ratio of net flow to reversibility magnitude. (**B**) Line plot showing a moving average (over a 30-cell window) of the proportion of cells in each cell cycle phase as a function of angle from the origin (angles beginning from zero on the positive *x* axis). Purple series shows the ratio of net flow to reversibility magnitude (tangential component only), scaled to a range of 0–1. (**C**) PCA projection of U2OS scRNA-seq data colored by the ratio of net flow to reversibility magnitude. (**D**) Line plot showing a moving average (over a 30-cell window) of the proportion of cells in each cell cycle phase as a function of angle from the origin (angles beginning from zero on the positive *x* axis). Purple series shows the ratio of net flow to reversibility magnitude (tangential component only), scaled to a range of 0–1.

 © The Author(s)

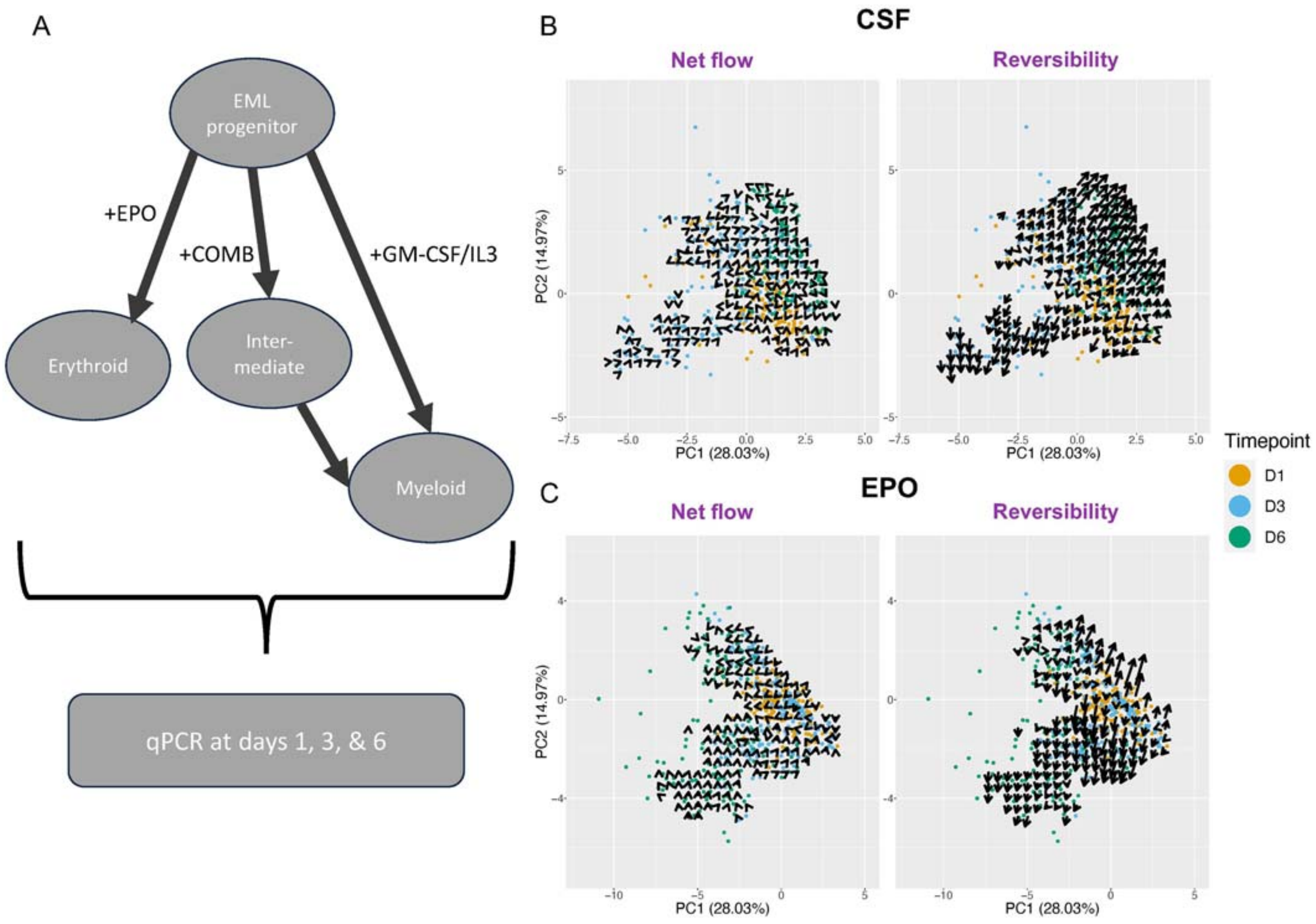

Figure EV4. STICCC results for HSC dataset separated by treatment.

(**A**) Diagram describing the experimental design for HSC dataset: EML progenitor cells were treated with EPO, GM-CSF/IL3, or a combination of both stimuli. (**B**) Net flow and reversibility predictions for HSC cells treated with CSF. Point color denotes timepoint. (**C**) Predictions for HSC cells treated with EPO.

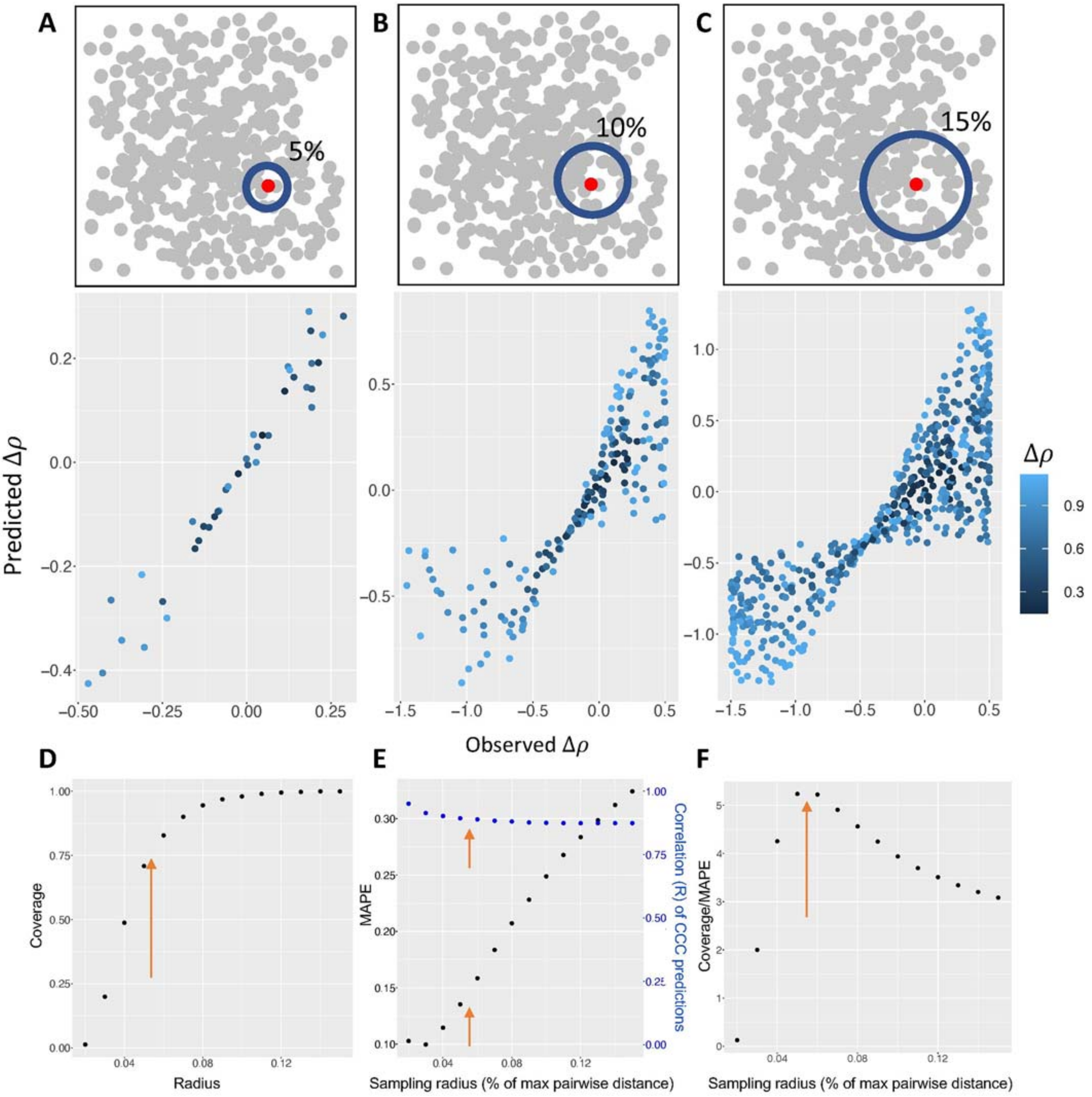

Figure EV5. Linear regression quality depends on sampling radius.

(**A–C**) Example neighborhoods for a sampling radius of 5%, 10%, and 15% of the maximum pairwise Euclidean distance in gene expression between cells, respectively, illustrated by a blue circle around a red center cell. Below, scatterplots show observed and predicted CCC values for neighboring cells. (**D**) Coverage, i.e., proportion of cells for which a prediction is generated, as a function of sampling radius. (**E**) Median absolute percent error (MAPE), left axis, and R value of linear regression, right axis, for various sampling radii. (**F**) Ratio of coverage to MAPE for various sampling radii.

 © The Author(s)
